# Changes in Central Macular Thickness following Single Session Multispot Panretinal Photocoagulation

**DOI:** 10.1155/2015/529529

**Published:** 2015-01-28

**Authors:** Nawat Watanachai, Janejit Choovuthayakorn, Direk Patikulsila, Nimitr Ittipunkul

**Affiliations:** Department of Ophthalmology, Faculty of Medicine, Chiang Mai University, Chiang Mai 50200, Thailand

## Abstract

*Purpose*. To determine changes in central subfield (CSF) macular thickness and best corrected visual acuity (BCVA) following single session, multispot panretinal photocoagulation (PRP). *Methods*. Forty eyes of 33 patients with newly diagnosed proliferative diabetic retinopathy were treated with single session, 20-millisecond, multispot PRP. Changes in central macular thickness and BCVA at 4- and 12-week follow-up were compared to baseline measurements. * Results*. Each eye received a mean (SD) of 2,750 (686.7) laser spots. At 4-week follow-up, there was a statistically significant 24.0 *μ*m increase in mean CSF thickness (*P* = 0.001), with a 17.4 *μ*m increase from baseline at 12-week follow-up (*P* = 0.002). Mean logMAR BCVA increased by 0.05 logMAR units (*P* = 0.03) at 4-week follow-up. At 12-week follow-up, BCVA had almost returned to normal with only an increase of 0.02 logMAR units compared to baseline (*P* = 0.39). Macular edema occurred in 2 eyes (5%) at 12-week follow-up. *Conclusions*. Macular thickening occurs following single session, 20-millisecond, multispot PRP, with a corresponding, mild change in BCVA. However, the incidence of macular edema appears to be low in these patients. Single session, 20-millisecond, multispot PRP appears to be a safe treatment for patients with proliferative diabetic retinopathy.

## 1. Introduction

Panretinal photocoagulation (PRP) is performed as a standard treatment for proliferative diabetic retinopathy (PDR) and is also frequently used to treat severe nonproliferative diabetic retinopathy. Its utility in reducing the risk of severe vision loss was demonstrated by the Diabetic Retinopathy Study and the Early Treatment Diabetic Retinopathy Study (ETDRS) [1–3]. The pathophysiologic changes leading to neovascularization regression following PRP remain uncertain. However, it is postulated that PRP decreases the metabolic activity of the ischemic retina, reduces the production of angiogenic factors, and improves inner retinal circulation [4]. Conventional PRP is delivered in one or more sessions in single spot mode, with the laser spot size ranging from 100 to 500 *μ*m and a pulse duration ranging from 100 to 200 milliseconds (ms). While the overall visual benefit of this therapy is not disputed, visual disabilities have been reported after conventional PRP, including central visual loss caused by the development of macular edema and peripheral visual loss from extensive, expanding inner retinal scarring [5–7].

In an attempt to minimize the drawbacks of conventional PRP, an innovative photocoagulator with the capability to deliver multispot, medium pulse duration laser burns was recently introduced into clinical practice [8]. Optimal laser parameters applied with 20 ms pulse duration PRP have been investigated at several centers [9–11]. Several studies have demonstrated comparable efficacy of multispot, 20 ms pulse duration PRP, delivered in either a single session or multiple sessions, to conventional, single spot 100 ms pulse duration PRP, in terms of regression of retinal neovascularization [12, 13]. In addition, 20 ms pulse duration PRP resulted in more uniformity of laser spots, less inner retinal tissue damage, and increased patient comfort compared to conventional 100 ms pulse duration PRP [14–18]. Single session, multispot, 20 ms pulse duration PRP is well tolerated by patients in part because it is less time-consuming and reportedly less painful. In addition, single session PRP reduces both direct and indirect hospital visit expenses for patients while simultaneously improving clinic efficiency for ophthalmologists. For these reasons, single session, multispot, 20 ms pulse duration PRP is increasingly being performed in clinical practice. However, the adverse effects of single session, multispot, 20 ms pulse duration PRP, including postlaser macular edema, need to be addressed. Unlike stereoscopic biomicroscopy, optical coherence tomography (OCT) is able to quantitatively evaluate subtle changes in macular thickness [19, 20] and has been used for objective assessment of macular thickness changes in both clinical practice and in numerous clinical trials.

The primary aim of this study is to evaluate the effect of single session, multispot, 20 ms pulse duration PRP on central macular thickness, as measured by spectral domain optical coherence tomography (SD-OCT). In addition, best corrected visual acuity (BCVA) changes and other complications related to PRP are reported to provide additional insight into this laser therapy delivery system.

## 2. Materials and Methods

The medical records of 35 patients presenting to the Department of Ophthalmology, Faculty of Medicine, Chiang Mai University, Thailand, from January 2012 to January 2013, with newly diagnosed, treatment naïve PDR, were retrospectively reviewed. The study was conducted according to the tenets of the Declaration of Helsinki. The hospital institutional review board and ethics committee approved the study protocol.

### 2.1. Patient Eligibility

Patients with type 1 or type 2 diabetes and newly diagnosed PDR were enrolled if they met the following inclusion criteria: (1) age ≥ 18 years; (2) BCVA of 70 ETDRS letters or better (Snellen equivalent of 6/12 or better); (3) central subfield (CSF) retinal thickness measured by SD-OCT ≤ 320 *μ*m with no structural abnormalities. CSF was defined as a circular area centered around the fovea with a diameter of 1,000 *μ*m. Exclusion criteria included the following: (1) history of prior treatments for diabetic macular edema (DME); (2) history of prior PRP; (3) history of intraocular surgery within the last 6 months; (4) presence of subretinal or intraretinal fluid or cystic changes on OCT in the CSF; (5) aphakia; (6) coexisting ocular diseases that could influence VA and macular thickness; (7) chronic renal failure requiring dialysis or kidney transplant; (8) systemic blood pressure (BP) more than 180/110 mmHg; (9) inadequate media clarity to perform complete laser in one session; (10) inadequate follow-up, defined as missing the 4- or 12-week follow-up visits.

### 2.2. Study Design

Patients' baseline characteristics were recorded including age, sex, type and duration of diabetes, glycosylated hemoglobin A1C level within the last 3 months, and disease laterality. All patients underwent a complete ophthalmic examination including BCVA, intraocular pressure (IOP) measurement using Goldmann applanation tonometer, slit-lamp biomicroscopy, and dilated fundus examination. Color fundus photography was performed with a fundus camera (KOWA VX-10i, Kowa, Tokyo, Japan) and retinal thickness measurement was performed with a SD-OCT (Spectralis; Heidelberg Engineering, CA, USA). The OCT image was scanned in a 20 × 20-degree high resolution volumetric pattern consisting of 49 horizontal B-scan lines with 1,024 A-scans per line. All scans were reviewed for accuracy by one investigator (Janejit Choovuthayakorn). The patients underwent follow-up at 4 weeks and 12 weeks (±7 days) after PRP. BCVA, IOP, slit-lamp biomicroscopy, fundus photography, and OCT imaging were performed at each follow-up visit. One masked investigator (Direk Patikulsila) reviewed the fundus photographs, comparing baseline and subsequent follow-up photos to assess for the regression of neovascularization.

### 2.3. Laser Photocoagulation Technique

PRP was performed with a 532 nm frequency-doubled neodymium-doped yttrium aluminium garnet (Nd-YAG) solid-state pattern scan laser (VALON pattern laser, Dual Laser Ltd. Oy, Vantaa, Finland) using a SuperQuad 160 contact lens (laser spot magnification of 2.0; Volk Optical Inc., Mentor, OH). A 4 × 4-multispot array with 200 *μ*m spot size, 20 ms pulse duration, and 1.5-width spot spacing was used. The burn intensity was titrated until a gray-white opacity was achieved. PRP was placed from just outside the vascular arcades to the peripheral retina, with care taken to prevent laser burns from encroaching within 2 disc diameters (DD) temporal to fovea or 1 DD nasal to the optic disc. All laser sessions were delivered under topical anesthesia (0.5% tetracaine hydrochloride ophthalmic solution) by one retinal specialist (Nawat Watanachai).

### 2.4. Statistical Analysis

Descriptive statistics were performed for patients' baseline characteristics and reported as mean (standard deviation, SD) or median (range) for continuous data and frequency (percentage) for category data. A paired *t*-test was used to compare the mean CSF thickness and BCVA in eyes at baseline and at each follow-up visit. Pearson's correlation was performed to evaluate the correlation of changes in CSF thickness and BCVA at each follow-up visit. Univariate logistic regression was used to evaluate the associations between changes in CSF thickness at 12-week follow-up and other parameters. BCVA was converted to a logarithm of the minimal angle of resolution (logMAR) units for statistical calculation. All analyses were based on a 2-tailed test of significance and performed by Stata version 12.0 (College Station, TX; StataCorp LP). A *P* < 0.05 was considered significant.

## 3. Results

Of the 35 patients who met all the eligibility criteria, 2 patients were excluded due to inadequate follow-up. Forty eyes of 33 consecutive patients were reviewed. The mean age (SD) of the patients was 52.3 (7.7) years. Of the 33 patients, 32 (97%) had type 2 diabetes and one (3%) had type 1 diabetes. Patients' baseline characteristics are shown in Table 1. For laser application, the mean total number (SD) of spots was 2,750 (686.7) with a mean laser power (SD) of 399 (116.8) mW delivered per eye. The laser parameters are shown in Table 2.

**Figure 1:**
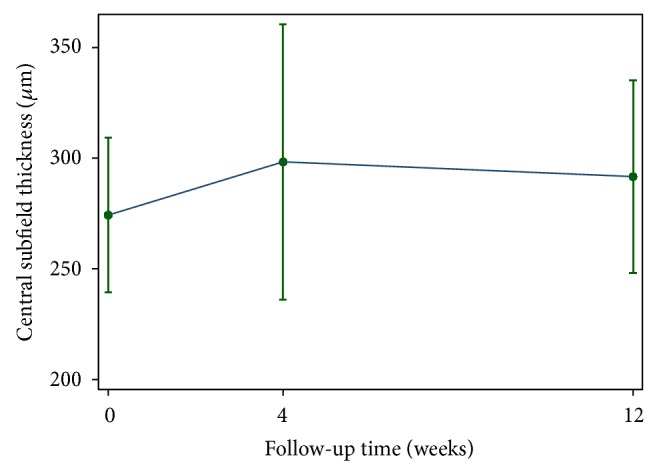
Changes in macular central subfield thickness after single session, multispot, 20-millisecond panretinal photocoagulation.

The mean CSF thickness (SD) at baseline was 274.3 (24.9) *μ*m. At 4-week follow-up, the mean CSF thickness increased by 24.0 *μ*m (95% CI; 12.8 to 35.1 *μ*m, *P* = 0.001) and at 12-week follow-up the CSF thickness increased by 17.4 *μ*m (95% CI; 6.2 to 28.6 *μ*m, *P* = 0.002). Mean CSF thickness at baseline and at given follow-up visits is shown in Figure 1. The mean BCVA (SD) at baseline was 0.13 (0.11) logMAR units. At 4-week follow-up, there was a statistically significant increase in mean BCVA to 0.18 logMAR units (95% CI of mean difference; 0.03 to 0.10 logMAR units, *P* = 0.03). However, at 12-week follow-up, the increase in mean BCVA was no longer statistically significant at 0.14 logMAR units (95% CI of mean difference; −0.01 to 0.03 logMAR units, *P* = 0.34). Changes in CSF thickness and BCVA at 4-week follow-up (Pearson's correlation coefficient: −0.58, *P* = 0.001) and at 12-week follow-up (Pearson's correlation coefficient: −0.31, *P* = 0.06) were inversely correlated; however, the correlations were minimal. The changes in BCVA did not well correlate with the degree of CSF thickness changes. No severe visual loss occurred in this study.

At 12-week follow-up, univariate analysis showed that age, baseline VA, CSF thickness, glycosylated HbA1C level, and laser parameters were not significantly associated with changes in CSF thickness (Table 3). Two of the 40 eyes (5%) developed clinically significant macular edema requiring treatment. Additional PRP was performed on 10 eyes (25%). No patient experienced exudative retinal detachment, choroidal detachment, or severe visual loss.

## 4. Discussion

The present study evaluated the effect of single session, multispot, 20 ms pulse duration PRP on CSF thickness and BCVA in patients with newly diagnosed PDR without center-involving macular edema as confirmed by OCT. In this cohort, there was a statistically significant increase in CSF thickness at both the 4- and 12-week follow-up, while BCVA returned close to baseline at the 12-week follow-up visit. However, macular edema requiring further treatment was noted in 5% of treated eyes at the 12-week visit. There were no other adverse effects related to PRP.

Conventional PRP is the standard treatment for patients with PDR, with well-documented evidence of reduction in severe visual loss [1, 3]. Nevertheless, visually significant macular edema following PRP has been noted on slit-lamp biomicroscopy [21]. The introduction and application of OCT in the clinical setting have allowed for the detection and quantification of subtle macular changes [20]. Macular OCT assessment following PRP has been subsequently studied [6, 22–24]. Those studies showed significant, mild macular thickening compared to baseline occurring after one or more sessions of conventional PRP. In these studies, macular edema either resolved or persisted throughout follow-up (ranging from 16 weeks to 12 months). However, an increase in macular thickness resulting in macular edema was reported in 9.7% to 15.7% of treated eyes, with follow-up ranging from 12 to 34 weeks. Pattern scan laser photocoagulators capable of delivering multispot arrays of laser at a reduced pulse duration of 20 ms have recently been introduced into clinical practice. The benefits of this laser delivery system include decreased time required to deliver treatment and improved patient comfort compared to conventional PRP [25, 26]. Consequently, many providers prefer multispot, 20 ms PRP to treat PDR. However, the effect of multispot, 20 ms PRP on macular thickness has been raised as a potential area of concern.

In the Manchester Pascal Study, Muqit et al. [14] conducted a randomized study comparing single spot, 100 ms, multisession PRP with a multispot, 20 ms single session PRP. In their cohort, 19 eyes received multispot, 20 ms, single session PRP. These patients developed a statistically insignificant 2 *μ*m increase in CSF thickness at 4-week posttreatment, with a statistically insignificant 2 *μ*m decrease at 12-week posttreatment. Macular edema was not observed in either arm of their study. However, the total number of laser spots performed in the eyes receiving multispot PRP was 1,500, comparable to the conventional PRP arm. Several studies have suggested that 1,500 laser spots may be insufficient for multispot, 20 ms PRP. Chappelow et al. [27] retrospectively studied 82 eyes with newly diagnosed, high-risk PDR who had at least 6 months of follow-up. They reported that eyes treated with multispot, 20 ms PRP exhibited a higher treatment failure rate, defined as either persistence or recurrence of neovascularization, than conventional PRP when delivered as a comparable number of laser spots. They hypothesized that the higher laser fluence of conventional, 100 ms PRP led to a larger area of heat diffusion and a larger area of coagulated retina following 100 ms conventional PRP, accounting for the difference in efficacy between these two laser parameters. Other studies have also shown that higher numbers of laser spots are required for multispot, 20 ms PRP to obtain a similar total retinal treatment area as that seen with 100 ms PRP [11, 13].

The present study showed a statistically significant increase in CSF thickness of 24 *μ*m and 17 *μ*m at 4- and 12-week follow-up, respectively, with an average of 2,750 laser spots delivered. The higher number of laser spots in the present study may explain the increased CSF thickening observed. Oh et al. [28] retrospectively reviewed 129 eyes after single session, multispot, 20 ms PRP. With a mean number of 3,125 laser spots delivered, they reported a statistically significant CSF thickness increase of 20 *μ*m at 1-month follow-up. At 12-week follow-up, 8.5% of the eyes in their study developed macular edema. In the present study, 5% of the eyes were noted to develop macular edema. Oh and colleagues used multivariate analysis to study risk factors for the development of macular edema following PRP. They found that a thickened macula and the presence of intraretinal cystoid spaces or subretinal fluid on baseline OCT were significant predictors for macular edema development following PRP. However, no patients in the present study had these features on pretreatment OCT, which may explain the slightly lower incidence of macular edema in this study. Our results confirm that careful posttreatment monitoring is required to detect the development of macular edema following multispot PRP, regardless of the presence of preexisting risk factors for the development of macular edema.

The effect of single-spot, 20 ms pulse duration PRP on macular edema has also been investigated. Mirshahi et al. [26] carried out a randomized study comparing single spot, 20 ms PRP with single spot, 100 ms PRP in 66 eyes. In the 20 ms PRP arm, with a mean of 2,125 laser spots, they reported a 13 *μ*m increase in CSF thickness at 1-month and 9 *μ*m increase at 4-month follow-up compared to baseline. The group receiving conventional, 100 ms PRP had a 53 *μ*m increase in CSF thickness at 1-month and a 50.8 *μ*m increase at 4-month follow-up compared to baseline, with a mean of 1,218 laser spots. Mirshahi and colleagues showed increasing post-PRP macular thickening in the single spot, 100 ms PRP group. Post-PRP macular thickening in the single spot, 20 ms PRP groups was similar to the present study; however, more time was required to deliver treatment in their cohort since they utilized single spot therapy. This data, combined with the findings of the present study, suggests that 20 ms PRP, whether delivered as multispot or single spot treatment, reduces CSF thickness following PRP. Multispot treatment has the advantage of reducing treatment time, though single spot, 20 ms treatment may be a good option for physicians who do not have access to a multispot laser system.

Previous studies have demonstrated that the range of repeatability and reproducibility of SD-OCT CSF thickness measurements in patients with diabetic macular edema is 8 to 12 *μ*m [29–31]. We performed a univariate analysis for CSF macular changes beyond 12 *μ*m to evaluate factors related to macular thickness changes. Only gender was found to correlate with increased CSF thickness. However, the sample size was not large enough to calculate a power association. Further study of factors associated with post-PRP macular edema development is needed.

With regards to the effect of PRP on BCVA, Muqit et al. [14] reported stabilized vision over 12-week follow-up in patients who received 1,500-spot, single session, multispot, 20 ms PRP. Oh et al. reported a significant increase in mean logMAR BCVA from 0.2 at baseline to 0.24 at both 1- and 3-month visits [28]. The present study shows a trend toward a statistically significant increase in mean logMAR BCVA from 0.13 to 0.18 at 4-week follow-up compared to baseline. BCVA then improved to 0.14 logMAR units at 12-week follow-up. The change in BCVA from baseline to 12-week follow-up was not statistically significant. A transient increase in logMAR BCVA may occur in the early phase following multispot, 20 ms, single session PRP, and subsequent improvement in BCVA to baseline level may be observed at 12-week follow-up. However, while the increase in logMAR BCVA at 4-week follow-up was statistically significant, it represented less than 1 line of vision and is likely not clinically meaningful.

This study is not without limitations, including those inherent to a retrospective study. Our findings, including the decreased incidence of macular edema compared to previous studies, may have been influenced by the small sample size. However, it is equally plausible that the decreased incidence of macular edema was secondary to the absence of pre-PRP risk factors for macular edema. Total follow-up for this study was short; however, it mirrors common clinical practice and as such provides valuable prognostic information to providers. When possible, our results were compared to those of other studies, though direct comparisons were limited by variation in study design.

In conclusion, patients treated with multispot, 20 ms PRP in a single session were shown to have slight macular thickening that persisted through 12-week follow-up. Two patients (5%) developed macular edema, which is slightly less common than previous reports. In addition, logMAR BCVA increased slightly at 4-week follow-up, improving to near baseline at 12-week follow-up. This study adds additional support to the use of single session, multispot, 20 ms PRP in common clinical practice. While rare, care should be taken in monitoring patients for the development of macular edema following single session, multispot PRP, as this can be vision threatening.

## Figures and Tables

**Table 1:** Baseline characteristics of patients undergoing single session, multispot, 20-millisecond, panretinal photocoagulation.

Characteristics	
Gender, number (%)	
Male	11 (33.3)
Female	22 (66.7)
Age (year), mean (SD)	52.3 (7.7)
Laterality, number (%)	
Right	22 (55.0)
Left	18 (45.0)
Type of diabetes, *n* (%)	
I	1 (3.0)
II	32 (97.0)
Duration of diabetes (year), median (min–max)	10 (0.2–25.0)
Glycosylated HbA1C (mg/dL), mean (SD)	9.2 (2.4)
Baseline logMAR BCVA, mean (SD)	0.13 (0.11)

SD: standard deviation, logMAR: logarithm of the minimal angle of resolution visual acuity.

**Table 2:** Laser parameters performed in single session, multispot, 20-millisecond panretinal photocoagulation.

Parameters	
Laser power (mW), mean (SD)	399 (116.8)
Laser fluence (J/cm^2^), median (range)	5.4 (4.7 to 36.9)
Total laser area (mm^2^), mean (SD)	402.0 (155.8)
Total laser energy, mean (SD)	21.7 (8.0)
Total laser burn (spots), mean (SD)	2,750.0 (686.7)
Duration of treatment (min), mean (SD)	4.9 (0.9)

mW: milliwatt, SD: standard deviation, J: Joule, and min: minute.

**Table 3:** Univariate analysis of factors related to changes incentral subfield thickness at 12-week follow-up after single session, multispot, 20-millisecond panretinal photocoagulation.

Characteristics mean (SD)	Changes in CSF thickness		*P* value
	≥12 *μ*m	<12 *μ*m	
Gender, *n* (%)			0.02
Male	3 (21.4)	11 (78.8)	
Female	16 (61.5)	10 (38.5)	
Age, year	52.58 (7.9)	52.0 (7.7)	0.82
Baseline logMAR BCVA, mean (SD)	0.13 (0.08)	0.14 (0.09)	0.57
Baseline CSF thickness, *μ*m	275.5 (36.5)	273.2 (34.3)	0.83
Baseline HbA1C, mg/dL	8.7 (2.2)	9.7 (2.4)	0.16
PRP parameters			
Laser power (mW)	380.0 (110.2)	420.0 (122.7)	0.36
Laser fluence (J/cm^2^)	5.4 (3.7)	5.4 (3.2)	0.84
Laser area (mm^2^)	369.4 (149.7)	431.4 (158.9)	0.21
Total laser energy	21.7 (7.2)	21.7 (8.8)	0.87
Total laser burn (spot)	2889.0 (776.1)	2625.0 (585.3)	0.78
Duration of PRP (min)	5.1 (0.9)	4.7 (0.7)	0.18

SD: standard deviation, VA: visual acuity, CSF: central subfield, mW: milliwatt, J: Joule, min: minute, and PRP: pan retinal photocoagulation.
